# Therapeutic targeting of erbB3 with MM-121/SAR256212 enhances antitumor activity of paclitaxel against erbB2-overexpressing breast cancer

**DOI:** 10.1186/bcr3563

**Published:** 2013-10-29

**Authors:** Shuiliang Wang, Jingcao Huang, Hui Lyu, Bo Cai, Xiaoping Yang, Fang Li, Jianming Tan, Susan M Edgerton, Ann D Thor, Choon-Kee Lee, Bolin Liu

**Affiliations:** 1 Department of Pathology, School of Medicine, University of Colorado Anschutz Medical Campus, MS-8104, 12801 E. 17th Ave., Aurora, CO 80045, USA; 2 Division of Medical Oncology, School of Medicine, University of Colorado Anschutz Medical Campus, Aurora, CO, USA; 3 Department of Dermatology, Duke University School of Medicine, Durham, NC, USA; 4 Fujian Key Laboratory of Transplant Biology, Fuzhou General Hospital, Xiamen University, Fuzhou, Fujian, China

## Abstract

**Introduction:**

Elevated expression of erbB3 rendered erbB2-overexpressing breast cancer cells resistant to paclitaxel via PI-3 K/Akt-dependent upregulation of Survivin. It is unclear whether an erbB3-targeted therapy may abrogate erbB2-mediated paclitaxel resistance in breast cancer. Here, we study the antitumor activity of an anti-erbB3 antibody MM-121/SAR256212 in combination with paclitaxel against erbB2-overexpressing breast cancer.

**Methods:**

Cell growth assays were used to determine cell viability. Cells undergoing apoptosis were quantified by a specific apoptotic ELISA. Western blot analyses were performed to assess the protein expression and activation. Lentiviral vector containing shRNA was used to specifically knockdown Survivin. Tumor xenografts were established by inoculation of BT474-HR20 cells into nude mice. The tumor-bearing mice were treated with paclitaxel and/or MM-121/SAR256212 to determine whether the antibody (Ab) enhances paclitaxel’s antitumor activity. Immunohistochemistry was carried out to study the combinatorial effects on tumor cell proliferation and induction of apoptosis *in vivo*.

**Results:**

MM-121 significantly facilitated paclitaxel-mediated anti-proliferative/anti-survival effects on SKBR3 cells transfected with a control vector or *erbB3* cDNA. It specifically downregulated Survivin associated with inactivation of erbB2, erbB3, and Akt. MM-121 enhances paclitaxel-induced poly(ADP-ribose) polymerase (PARP) cleavage, activation of caspase-8 and -3, and apoptosis in both paclitaxel-sensitive and -resistant cells. Specific knockdown of Survivin in the trastuzumab-resistant BT474-HR20 cells dramatically enhanced paclitaxel-induced apoptosis, suggesting that increased Survivin caused a cross-resistance to paclitaxel. Furthermore, the studies using a tumor xenograft model-established from BT474-HR20 cells revealed that either MM-121 (10 mg/kg) or low-dose (7.5 mg/kg) paclitaxel had no effect on tumor growth, their combinations significantly inhibited tumor growth *in vivo*. Immunohistochemical analysis showed that the combinations of MM-121 and paclitaxel significantly reduced the cells with positive staining for Ki-67 and Survivin, and increased the cells with cleaved caspase-3.

**Conclusions:**

The combinations of MM-121 and paclitaxel not only inhibit tumor cell proliferation, but also promote erbB2-overexpressing breast cancer cells to undergo apoptosis via downregulation of Survivin *in vitro* and *in vivo*, suggesting that inactivation of erbB3 with MM-121 enhances paclitaxel-mediated antitumor activity against erbB2-overexpressing breast cancers. Our data supports further exploration of the combinatorial regimens consisting of MM-121 and paclitaxel in breast cancer patients with erbB2-overexpressing tumors, particularly those resistant to paclitaxel.

## Introduction

The erbB receptor tyrosine kinase (RTK) family, including the epidermal growth factor receptor (EGFR), erbB2 (or Her2/neu), erbB3, and erbB4, is arguably the most important receptor family in the context of development and tumorigenesis [1,2]. Amplification and/or overexpression of e*rbB2* occur in approximately 25 to 30% of invasive breast cancers and are significantly associated with a worse prognosis in breast cancer patients [3,4]. Numerous studies indicate that increased treatment resistance and enhanced metastatic potential are two of the major mechanisms by which erbB2 contributes to breast carcinogenesis [5,6]. Most metastatic breast cancers show expression for either EGFR or erbB2, and less often for both [7]. In contrast, co-expression of erbB2 and erbB3 frequently occurs in breast cancers [8] and breast cancer cell lines [9]. The erbB3 receptor is unique among the four erbB family members. Unlike EGFR, erbB2, and erbB4, it lacks kinase activity [10,11] or possesses weak kinase activity [12]. However, erbB3 has been shown to serve as a critical co-receptor of erbB2, and its expression is a rate-limiting factor for erbB2-mediated breast cancer cell survival and proliferation [13,14]. We and others have also observed an elevated expression of the endogenous mouse erbB3 in the mammary tumors derived from *erbB2*/*neu*-transgenic mice [15,16]; and the increased erbB3 forms physical and functional interactions with the transgene-encoded erbB2 to promote mammary tumorigenesis [15]. In fact, the erbB2/erbB3 heterodimer has been identified as the most potent form of all erbB receptor complexes to activate the oncogenic signaling, such as PI-3 K/protein kinase B (Akt), mitogen-activated protein kinase kinase (MEK)/mitogen-activated protein kinase (MAPK), and/or janus kinase (Jak)/signal transducer and activator of transcription (Stat) pathways, and/or Src kinase, in breast cancers [17-19].

Mechanistic studies implicate the function of erbB3 as a major cause of treatment failure in human cancers [20]. Therapeutic targeting of erbB3 is being investigated. Currently, no erbB3-targeted therapy has been approved for cancer treatment. Several erbB3 blocking antibodies (Abs) that prevent ligand-induced activation of erbB3, such as MM-121/SAR256212 (which is being co-developed by Merrimack Pharmaceuticals, Cambridge, MA, USA and Sanofi, Cambridge, MA, USA), MM-111 (Merrimack Pharmaceuticals) and U3-1287/AMG 888 (Amgen Inc., Thousand Oaks, CA, USA) are actively under preclinical and clinical studies [21] and show significant antitumor activity in preclinical studies [22-25]. MM-121 is a fully humanized anti-erbB3 monoclonal IgG2 Ab. It inhibits ligand-induced activation of erbB3, and exerts antitumor activity in human cancer models [23,24]. Nonetheless, MM-121’s therapeutic potential against erbB2-overexpressing breast cancers that are resistant to chemotherapy and/or the erbB2-targeted therapy has not been explored.

We have reported that activation of erbB3, mainly through PI-3 K/Akt signaling, plays a critical role in erbB2-mediated therapeutic resistance to tamoxifen [26] and paclitaxel [27]. The erbB3 receptor also interacts with both erbB2 and the insulin-like growth factor-1 receptor (IGF-1R) to form a heterotrimeric complex, which activates the PI-3 K/Akt signaling and Src kinase and subsequently results in resistance to erbB2-targeted therapy, trastuzumab (Herceptin) [28]. Since activation of the PI-3 K/Akt signaling is the major determinant of treatment resistance [29], we investigated, *in vitro* and *in vivo*, whether the anti-erbB3 Ab MM-121 would be able to overcome resistance and enhance the efficacy of chemotherapy or trastuzumab against erbB2-overexpressing breast cancer models via inhibition of the erbB3/PI-3 K/Akt signaling. In the current report, we sought to determine the antitumor activity of MM-121 in combination with paclitaxel against erbB2-overexpressing breast cancer using both *in vitro* and *in vivo* models. Our previous studies indicated that elevated expression of erbB3 led to paclitaxel resistance in erbB2-overexpressing breast cancer cells via PI-3 K/Akt signaling-dependent upregulation of Survivin [27]. Thus, we have focused on studying whether inactivation of erbB3 signaling with MM-121 may specifically downregulate Survivin, and subsequently re-sensitize the otherwise resistant breast cancer cells to paclitaxel-mediated anti-proliferative/anti-survival effects and apoptosis.

## Methods

### Reagents and antibodies

MM-121 was from Merrimack Pharmaceuticals, Inc.. Paclitaxel (Ben Venue Labs, Inc., Bedford, OH, USA) was obtained from University of Colorado Hospital pharmacy. Antibodies used for western blots were as follows: erbB2 (EMD Chemicals, Inc., Gibbstown, NJ, USA); erbB3 and P-erbB2 (Tyr1248) (LabVision Corp., Fremont, CA, USA); P-erbB3 (Tyr1289), caspase-8 (1C12), and caspase-3 (8G10), P-MAPK (Thr202/Tyr204), MAPK, P-Akt (Ser473), Akt, Survivin (6E4), Bcl-xL (Cell Signaling Technology, Inc., Beverly, MA, USA); Mcl-1 (clone 22) (BD Biosciences, San Jose, CA, USA); poly(ADP-ribose) polymerase (PARP) (BIOMOL Research Laboratories Inc., Plymouth Meeting, PA, USA); and β-actin (Sigma-Aldrich Co., St. Louis, MO, USA). All other reagents were purchased from Sigma unless otherwise specified.

### Cells and cell culture

Human breast cancer cell lines MCF-7, MDA-MB-231, SKBR3, and BT474 were obtained from the American Type Culture Collection (Manassas, VA, USA). The SKBR3.B3.1 and SKBR3.B3.2 cells are two *erbB3*-transfected stable clones, and the SKBR3.neo1 is an empty vector-transfected clone of SKBR3 cells [27]. The trastuzumab-resistant sublines BT474-HR20 and SKBR3-pool2, derived from BT474 and SKBR3, respectively, were described previously [28]. All cell lines were maintained in DMEM/F-12 (1:1) (Sigma) containing 10% FBS (Sigma), and cultured in a 37°C humidified atmosphere containing 95% air and 5% CO_2_ and split twice a week.

### Cell proliferation assay

The CellTiter96 AQ nonradioactive cell proliferation kit (Thermo Fisher Scientific Inc., Waltham, MA, USA) was used to determine cell viability as previously described [27,28,30]. Briefly, cells were plated onto 96-well plates for 24 h, and then grown in either DMEM/F12 medium with 0.5% FBS as control, or the same medium containing different concentrations of paclitaxel in the presence or absence of MM-121, and then incubated for another 72 h. After reading all wells at 490 nm with a microplate reader, the percentages of surviving cells from each group relative to controls, defined as 100% survival, were determined by reduction of 3-(4,5-dimethylthiazol-2-yl)-5-(3-carboxymethoxyphenyl)-2-(4-sulfophenyl)-2H-tetrazolium, inner salt (MTS).

### Specific knockdown of Survivin expression with a lentiviral system

Lentiviral production and specific knockdown of Survivin expression with an shRNA strategy were carried out as described previously [27]. In brief, the lentivirus-containing either control shRNA or Survivin specific shRNA (Survivin-S3 or Survivin-S5) were produced in 293 T cells following the standard procedure. The virus in conditioned medium were harvested, aliquoted, and stored in a -80°C freezer. Prior to infection, the lentivirus-containing media were thawed completely at room temperature, and mixed with the same amount of fresh medium containing polybrene (8 μg/ml). The culture media of the candidate breast cancer cells were then replaced with the lentivirus-containing media. After 24 h, the virus-infected cells were selected with puromycin (1 μg/ml) for 48 h, and then subjected to required experiments.

### Quantification of apoptosis

An apoptotic ELISA kit (Roche Diagnostics Corp., Indianapolis, IN, USA) was used to quantitatively measure cytoplasmic histone-associated DNA fragments (mononucleosomes and oligonucleosomes) as previously reported [27,28,30]. This enzyme immunoassay was performed according to the manufacturer’s instructions.

### Western blot analysis

Protein expression levels were determined by western blot analysis as described previously [27,28,30,31]. Equal amounts of total cell lysates were boiled in Laemmli SDS-sample buffer, resolved by SDS-PAGE, transferred to nitrocellulose membrane (Bio-Rad Laboratories, Hercules, CA, USA), and probed with the primary antibodies described in the figure legends. After the blots were incubated with horseradish peroxidase-labeled secondary antibody (Jackson ImmunoResearch Laboratories, West Grove, PA, USA), the signals were detected using the enhanced chemiluminescence reagents (GE Healthcare Bio-Sciences Corp., Piscataway, NJ, USA).

### Immunohistochemistry (IHC)

Five-micron-thick paraffin sections were deparaffinized, antigens unmasked and immunohistochemically stained for Ki-67 (Thermo Fisher Scientific; rabbit monoclonal SP6; cat# RM-9106-SO; dilution 1:500 in Tris-buffered saline and Tween 20 (TBST) + 1% BSA w/v), cleaved caspase-3 (Cell Signaling Technology; rabbit polyclonal; cat#: 9661, 1:1,000 in TBST + 1% BSA w/v), Survivin (Epitomics, Burlingame, CA, USA; rabbit monoclonal EP2880Y; cat# 2463; dilution 1:100 in TBST + 1% BSA w/v), erbB2 (EMD Chemicals; mouse monoclonal 96G; cat#OP14T; dilution 1:500 in TBST + 1% BSA w/v), and erbB3 (Spring Bioscience, Pleasanton, CA, USA; rabbit monoclonal SP71; cat# M3710; dilution 1:200 in TBST + 1% BSA w/v). The specificity of all antibodies has been confirmed by both positive and negative controls. For erbB2 and erbB3, SKBR3 cells were used as a positive control. For Survivin, the endometrial cancer tissues originating from ovary were used a positive control. For Ki-67 and cleaved caspase-3, human tonsil tissues were used a positive control. For the negative controls, in addition to use the same cells/tissues without addition of the primary antibodies, we also performed the IHC assays using the tissues or cell lines that are known to have no expression of the antigens (Additional file 1).

Ki-67, cleaved caspase-3 and Survivin antigens were revealed in pH 9.5 BORG solution (Biocare Medical, Concord, CA) for 5 minutes at 125°C (22 psi; decloaking chamber, Biocare). ErbB2 required modest retrieval in 10 mmol/L sodium citrate for 5 minutes at 125°C in the decloaking chamber. ErbB3 required retrieval in Cell Conditioner 1 (standard retrieval time, Ventana Medical Systems, Tucson, AZ, USA). Immunodetection of Ki-67, cleaved caspase-3 and erbB2 was performed on the NexES stainer (Ventana) at an operating temperature of 37°C. Ki-67 and cleaved caspase-3 antibodies were incubated for 32 minutes and detected with a modified I-VIEW DAB detection kit (Ventana). The I-VIEW secondary antibody and enzyme were replaced with a species-specific secondary antibody (biotinylated goat anti-rabbit; 1:75; cat# 111-065-144; Jackson ImmunoResearch; 8 minutes) and streptavidin-horseradish (SA-HRP; 1:50; cat# SA-5004; DAKO Cytomation, Carpinteria, CA, USA; 8 minutes). Survivin was optimized under ambient conditions with the Rabbit ImmPress polymer detection system (Vector Laboratories, Burlingame, CA, USA; cat# MP-7401). ErbB2 was incubated for 32 minutes and detected with the standard I-VIEW detection. ErbB3 was incubated for 32 minutes and detected with a modified I-VIEW DAB kit in which the secondary antibody was replaced with Rabbit ImmPress (Vector Laboratories; cat# MP-7401; 8 minutes at 37°C) and enzyme was replaced with Rabbit ImmPress (diluted 1:1 in PBS, pH 7.6; 8 minutes at 37°C). Sections were sequentially blocked for 10 minutes in 3% hydrogen peroxide (v/v) and 30 minutes in Rodent Block M (Biocare; cat# RBM961), followed by primary antibody incubation for 30 minutes and 30 minutes in polymer. Antibody complexes were visualized with IP Flex DAB (Biocare; cat# IPK5010 G80; 4.5%). All sections were counterstained in Mayer’s hematoxylin for 2 minutes, nuclei blued in 1% ammonium hydroxide (v/v), dehydrated in graded alcohols, cleared in xylene and coverglass-mounted using synthetic resin.

### Tumor xenograft model

Athymic nu/nu mice (Harlan Laboratories, Inc., Indianapolis, IN, USA) were maintained in accordance with the Institutional Animal Care and Use Committee (IACUC) procedures and guidelines approved by University of Colorado Health Sciences Center Animal Care and Use Committee. We suspended 8 × 10^6^ BT474-HR20 cells in 100 μL of PBS mixed with 50% Matrigel (BD Biosciences) and injected these subcutaneously into the flanks of 5-week-old female mice. Tumor formation was assessed by palpation and measured with fine calipers three times a week. Tumor volume was calculated by the formula shown below, where length was the longest axis and width the measurement at a right angle to the length:

Volume = (Length × Width^2^)/2

This was followed by statistical analysis as we described previously [32]. When tumors reached approximately 150 mm^3^ or 100 mm^3^, mice were randomly assigned into four groups (n = 5): 1) control-group mice received intraperitoneal (i.p.) injection of 100 μl PBS; 2) mice received i.p. injection of paclitaxel (15 mg/kg or 7.5 mg/kg) in 100 μl PBS twice a week; 3) mice received i.p. injection of MM-121 (10 mg/kg) in 100 μl PBS twice a week, or 4) mice received i.p. injection of paclitaxel (15 mg/kg or 7.5 mg/kg) and MM-121 (10 mg/kg) in 100 μl PBS twice a week. The animals’ health status was monitored daily for weight loss or for signs of altered motor ability while in their cages. At the end of study, mice were euthanized according to the approved IACUC protocol. Tumors from all animals were excised and embedded in paraffin for IHC analyses.

### Statistics

Statistical analyses of the experimental data were performed using either the two-sided *t*-test or analysis of variance (ANOVA) for each time point followed by post-hoc testing between groups. Significance was set at a *P*-value <0.05. All statistical analyses were conducted with the software StatView v5.1 from SAS Institute Inc., Cary, NC, USA.

## Results

### MM-121 overcomes paclitaxel resistance induced by co-expression of erbB2 and erbB3 in breast cancer cells and significantly enhances inhibitory activity of paclitaxel

**Figure 1:**
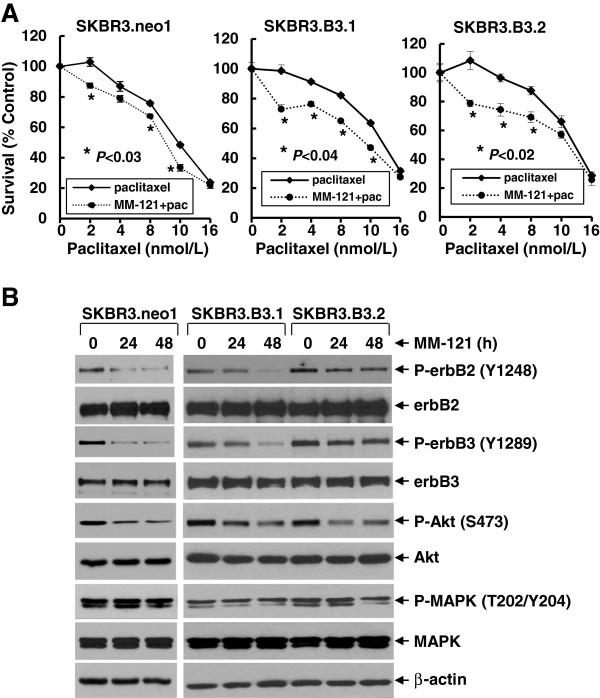
**MM-121 significantly enhances paclitaxel-mediated anti-proliferative/anti-survival effects on breast cancer cell lines with expression of both erbB2 and erbB3. (A)** SKBR3.neo1, SKBR3.B3.1, or SKBR3.B3.2 cells were plated onto 96-well plates and incubated at 37°C with 5% CO_2_. After 24 h, the culture medium was replaced with 0.1 ml fresh medium containing 0.5% FBS or the same medium containing the indicated concentrations of paclitaxel in the absence (paclitaxel) or presence (MM-121 + pac) of MM-121 (10 μg/ml) for another 72 h. The percentages of surviving cells from each cell line relative to controls, defined as 100% survival, were determined by reduction of 3-(4,5-dimethylthiazol-2-yl)-5-(3-carboxymethoxyphenyl)-2-(4-sulfophenyl)-2H-tetrazolium, inner salt (MTS). *Bars* represent SD. Data are representative of three independent experiments. **(B)** The same cells were untreated or treated with MM-121 (10 μg/ml) for 24 or 48 h. Cells were collected and subjected to western blot analyses of phosphorylated (P)-erbB2, erbB2, P-erbB3, erbB3, P-Akt, Akt, P-mitogen-activated protein kinase (P-MAPK), MAPK, or β-actin.

To study whether inactivation of erbB3 signaling with MM-121 may overcome paclitaxel resistance and facilitate paclitaxel-mediated inhibitory activity against erbB2-overexpressing breast cancer, the SKBR3.B3.1 and SKBR3.B3.2 cells that show resistance to paclitaxel due to ectopic expression of erbB3 in SKBR3 cells [27] were used to investigate inhibitory effects of MM-121 on erbB3 signaling and enhancement of paclitaxel-mediated anti-proliferative/anti-survival effects. Although SKBR3.B3.1 and SKBR3.B3.2 cell lines were less sensitive to paclitaxel than the parental SKBR3 and vector control SKBR3.neo1 cell lines [27], (this lower responsiveness was further confirmed in the current studies shown in Additional file 2), paclitaxel was still able to inhibit proliferative/survival of SKBR3.neo1, SKBR3.B3.1, and SKBR3.B3.2 cells in a dose-dependent manner (Figure 1A). Importantly, the presence of MM-121 significantly enhanced paclitaxel-mediated inhibitory activity in all three cell lines (Figure 1A). Western blot analyses showed that treatment of SKBR3.neo1, SKBR3.B3.1, and SKBR3.B3.2 cell lines with MM-121 reduced the levels of phosphorylated erbB3 (P-erbB3) and phosphorylated erbB2 (P-erbB2) in a time-dependent manner (Figure 1B). These data suggest that MM-121 mainly inhibits activation of erbB3, which is in agreement with other reports [23,24]. Since erbB3 interacted with the erbB2 receptor to activate erbB2, it is understandable that inactivation of erbB3 with MM-121 might disrupt the heterodimerization of erbB2/erbB3 receptors, and thus decrease the kinase activity of erbB2 (phosphorylation). Furthermore, treatment with MM-121 gradually reduced the levels of phosphorylated Akt (P-Akt), but had no significant effects on phosphorylated MAPK (P-MAPK) (Figure 1B). These data are consistent with our previous findings showing that the PI-3 K/Akt pathway is the major downstream signaling mechanism for erbB2/erbB3 interactions in breast cancer cell lines [26,27]. Taken together, our data indicate that the anti-erbB3 Ab MM-121 exhibits the potential to overcome paclitaxel resistance and significantly enhances paclitaxel-mediated anti-proliferative/anti-survival effects in erbB2-overexpressing breast cancer cell lines, with medium and high erbB3 expression, associated with its inhibition of the erbB3/PI-3 K/Akt signaling.

### MM-121 specifically downregulates Survivin in paclitaxel-resistant breast cancer cell lines and significantly enhances paclitaxel-induced apoptosis in both sensitive and resistant cells

**Figure 2:**
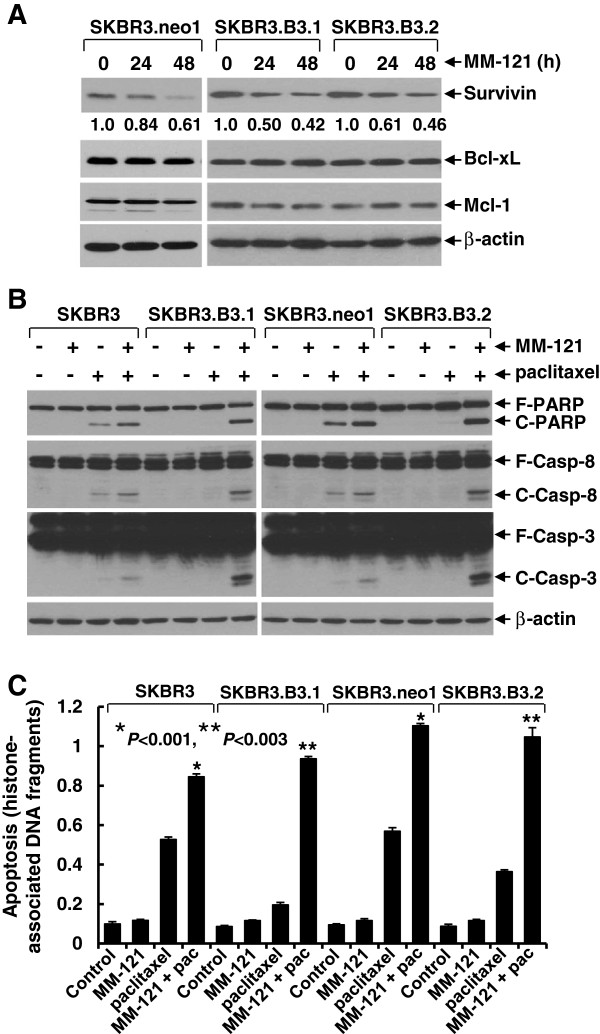
**MM-121 specifically downregulates Survivin and significantly enhances paclitaxel-induced apoptosis in paclitaxel-sensitive and -resistant breast cancer cells. (A)** SKBR3.neo1, SKBR3.B3.1, or SKBR3.B3.2 cells were untreated, or treated with MM-121 (10 μg/ml) for 24 or 48 h. Cells were collected and subjected to western blot analyses of Survivin, Bcl-xL, Mcl-1, or β-actin. The densitometry analyses of Survivin signals are shown underneath, and the arbitrary numbers indicate the intensities of each cell line relative to controls, defined as 1.0. **(B and C)** SKBR3 and SKBR3.B3.1, or SKBR3.neo1 and SKBR3.B3.2 cells were untreated, or treated with either MM-121 (10 μg/ml) or paclitaxel (3 nmol/L) alone, or their combinations for 24 h. Cells were collected (samples of SKBR3 and SKBR3.B3.1 cells were run on one gel, and samples from SKBR3.neo1 and SKBR3.B3.2 cells were run on another) and subjected to western blot analyses of poly(ADP-ribose) polymerase (PARP: F-PARP, full-length PARP; C-PARP, cleaved PARP), caspase-8 (F-Casp-8, full-length caspase-8; C-Casp-8, cleaved caspase-8), caspase-3 (F-Casp-3, full-length caspase-3; C-Casp-3, cleaved caspase-3), or β-actin **(B)**; or a specific apoptosis ELISA **(C)**. *Bars* represent SD. ^*^P <0.001 and ^**^P <0.003 versus single-agent treatment.

To study the molecular mechanism by which MM-121 overcomes paclitaxel resistance and increases paclitaxel-mediated anti-proliferative/anti-survival effects in the studied erbB2-overexpressing breast cancer cell lines, we investigated whether MM-121 might enhance paclitaxel-induced apoptosis. Because activation of erbB2/erbB3 signaling resulted in paclitaxel resistance via PI-3 K/Akt-dependent upregulation of Survivin [27] and we showed that MM-121 mainly inactivated Akt in all three SKBR3 sublines (Figure 1B), we hypothesized that MM-121 might inhibit Survivin expression in both paclitaxel-sensitive and -resistant breast cancer cells. Indeed, western blot analyses revealed that treatment of SKBR3.neo1, SKBR3.B3.1, and SKBR3.B3.2 cell lines with MM-121 led to decreased Survivin, but had no effects on the functionally related proteins, such as Bcl-xL and Mcl-1, in a time-dependent manner (Figure 2A). These data are consistent with our previous studies indicating that the erbB3 signaling specifically regulates Survivin expression in erbB2-overexpressing breast cancer cells [27]. Moreover, although SKBR3.B3.1 and SKBR3.B3.2 cells were less responsive than the parental SKBR3 and vector control SKBR3.neo1 cells to paclitaxel-induced apoptosis, the addition of MM-121 significantly enhanced paclitaxel-induced apoptosis in both paclitaxel-sensitive and -resistant breast cancer cells (Figure 2B and C). We showed evidence that the combinations of MM-121 and paclitaxel, as compared to either agent alone, gave rise to a profound induction of cleaved PARP and activation of caspase-8 and -3 (Figure 2B), as well as histone-associated DNA fragments (Figure 2C). Thus, our studies demonstrate that MM-121 overcomes paclitaxel resistance and enhances paclitaxel-induced apoptosis in the studied erbB2-overexpressing breast cancer cell lines via specific downregulation of Survivin.

### Elevated expression of Survivin is observed in one trastuzumab-resistant breast cancer cell line and shows cross-resistance to paclitaxel that can be abrogated by MM-121

**Figure 3:**
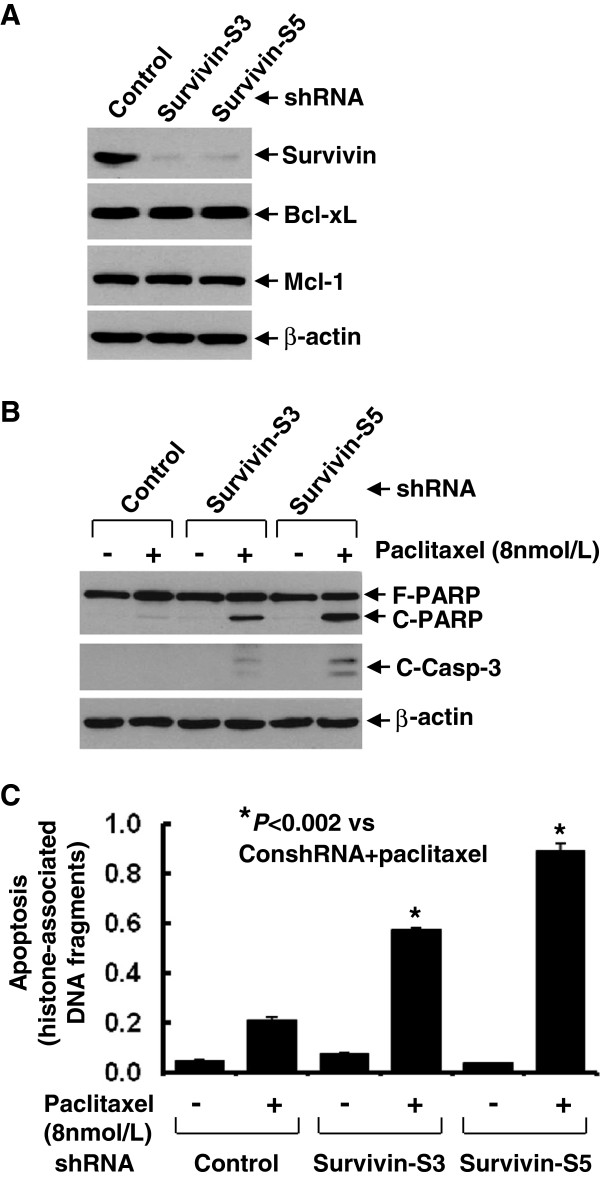
**Specific knockdown of Survivin expression with shRNA significantly promotes paclitaxel-induced apoptosis in a trastuzumab-resistant breast cancer cell line.** BT474-HR20 cells infected with lentivirus containing either control shRNA (ConshRNA) or Survivin shRNA (Survivin-S3, Survivin-S5) were subjected to the following experiments. **(A)** Western blot analyses of Survivin, Bcl-xL, Mcl-1, or β-actin. **(B and C)** The cells were then untreated, or treated with paclitaxel (8 nmol/L) for 24 h. The cells were collected and subjected to **(B)** western blot analyses of poly(ADP-ribose) polymerase (PARP), cleaved caspase-3, or β-actin; or to **(C)** apoptosis ELISA. *Bars* represent SD.

**Figure 4:**
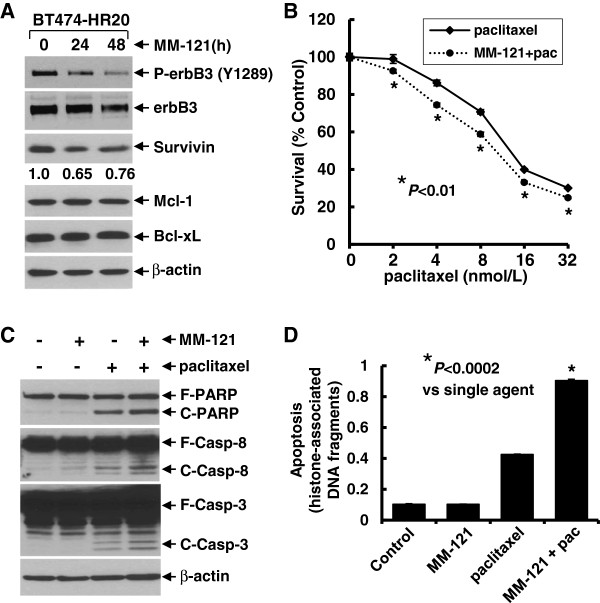
**MM-121 specifically downregulates Survivin and significantly enhances paclitaxel-induced anti-proliferative/anti-survival effects and apoptosis in a trastuzumab-resistant breast cancer cell line. (A)** BT474-HR20 cells were untreated, or treated with MM-121 (10 μg/ml) for 24 or 48 h. Cells were collected and subjected to western blot analyses of P-erbB3, erbB3, Survivin, Bcl-xL, Mcl-1, or β-actin. The densitometry analyses of Survivin signals are shown underneath, and the arbitrary numbers indicate the intensities of each sample relative to control, defined as 1.0. (**B)** BT474-HR20 cells were plated onto 96-well plates and incubated at 37°C with 5% CO_2_. After 24 h, the culture medium was replaced with 0.1 ml fresh medium containing 0.5% FBS or the same medium containing the indicated concentrations of paclitaxel in the absence (paclitaxel) or presence (MM-121 + pac) of MM-121 (10 μg/ml) for another 72 h. The percentages of surviving cells from each cell line relative to controls, defined as 100% survival, were determined by reduction of 3-(4,5-dimethylthiazol-2-yl)-5-(3-carboxymethoxyphenyl)-2-(4-sulfophenyl)-2H-tetrazolium, inner salt (MTS). *Bars* represent SD. Data are representative of three independent experiments. **(C and D)** BT474-HR20 cells were untreated, or treated with either MM-121 (10 μg/ml) or paclitaxel (8 nmol/L) alone, or their combinations for 24 h. Cells were collected and subjected to western blot analyses of poly(ADP-ribose) polymerase (PARP), caspase-8, caspase-3, or β-actin **(C)**; or a specific apoptosis ELISA **(D)**. *Bars* represent SD. ^*^P <0.0002 versus single-agent treatment.

We previously reported that the three RTKs, erbB2, erbB3, and IGF-1R interacted with each other to form a heterotrimeric complex, which activates the downstream signaling, such as PI-3 K/Akt or MEK/MAPK pathways and Src kinase in trastuzumab-resistant breast cancer cells [28]. We explored whether the trastuzumab-resistant sublines BT474-HR20 and SKBR3-pool2 might also have increased expression of Survivin due to the activation of PI-3 K/Akt signaling, and subsequently exhibit resistance to paclitaxel-induced apoptosis. Compared to the parental BT474 cells, the trastuzumab-resistant BT474-HR20 cells expressed much higher levels of Survivin and had a minor increase in Mcl-1. The expression levels of Bcl-xL showed no difference between BT474 and BT474-HR20 cells (Additional file 3: Figure S2A). Interestingly, BT474-HR20 cells were significantly more resistant to paclitaxel-mediated anti-proliferative/anti-survival effects than BT474 cells (Additional file 3: Figure S2B). However, this phenomenon was not observed in another pair of trastuzumab-sensitive SKBR3 and -resistant SKBR3-pool2 cells, as we did not find a significant induction of Survivin, Mcl-1, or Bcl-xL in SKBR3-pool2 cells (data not shown). The difference might be due to the fact that BT474-HR20 cells exhibited a dramatic activation of Akt as compared to BT474 cells, whereas significant activation of MAPK, but not Akt was discovered in SKBR3-pool2 cells [28]. To study whether the enhanced expression of Survivin in BT474-HR20 cells causally induced paclitaxel resistance, two shRNA sequences (Survivin-S3 and -S5) were used to specifically knock down Survivin (Figure 3A). Compared to control shRNA, both Survivin-S3 and Survivin-S5 significantly enhanced paclitaxel-induced apoptosis evidenced by increased PARP cleavage, caspase-3 activation (Figure 3B), and an apoptotic ELISA (Figure 3C). These data strongly suggest that the increased Survivin in the studied trastuzumab-resistant cell line causally elicited cross-resistance to paclitaxel. Because activation of the erbB3 signaling played an important role in the development of BT474-HR20 cells, we next studied whether MM-121 might also reduce Survivin in BT474-HR20 cells and therefore re-sensitize the cells to paclitaxel-mediated inhibitory activity and apoptosis. We discovered that MM-121 not only reduced the levels of P-erbB3, but also specifically downregulated Survivin (not Bcl-xL and Mcl-1) in BT474-HR20 cells (Figure 4A), consistent with our findings in the SKBR3 sublines (Figures 1B and 2A). More importantly, MM-121 significantly enhanced both paclitaxel-mediated anti-proliferative/anti-survival effects (Figure 4B) and apoptosis (Figure 4D), and facilitated paclitaxel-induced PARP cleavage and activation of caspase-8 and -3 (Figure 4C) in BT474-HR20 cells. Collectively, our data demonstrate that MM-121 was also able to abrogate paclitaxel resistance-induced by elevated expression of Survivin in the trastuzumab-resistant breast cancer cells.

### The combinations of MM-121 and paclitaxel at a lower dose significantly inhibit tumor growth in a xenograft model established from the trastuzumab-resistant BT474-HR20 breast cancer cells

**Figure 5:**
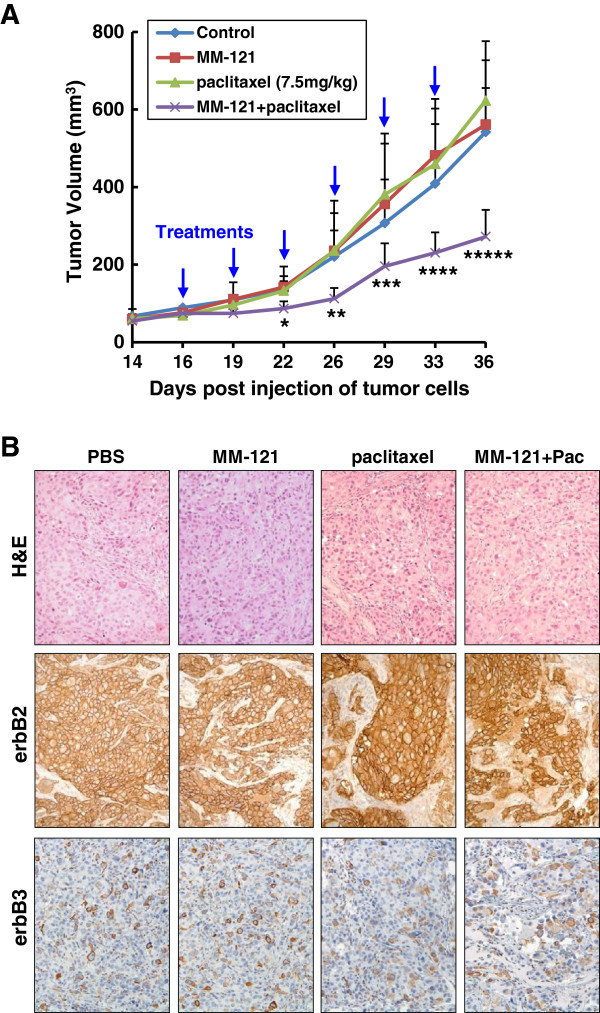
**MM-121 in combination with low-dose paclitaxel significantly inhibits *in vivo *tumor growth of trastuzumab-resistant breast cancer cells in a murine model.** BT474-HR20 cells were subcutaneously injected into nude mice to establish tumor xenografts. The tumor-bearing mice (n = 5) received intraperitoneal injections of either PBS, or MM-121 (10 mg/kg), or paclitaxel (7.5 mg/kg) alone, or both MM-121 and paclitaxel as described in Methods. After six treatments the mice were euthanized at day 36 post injection of tumor cells, and all tumors were excised for histology and IHC analysis. **(A)** The graphs show the tumor growth curves. *Bars* represent SD. ^*^P <0.04 versus control or MM-121; ^**^P <0.05 versus MM-121 and *P* <0.005 versus paclitaxel; ^***^P <0.04 versus paclitaxel; ^****^P <0.05 versus control, *P* <0.008 versus MM-121, and *P* <0.02 versus paclitaxel; ^*****^P = 0.005 versus control, *P* <0.004 versus MM-121, and *P* = 0.0006 versus paclitaxel. **(B)** Data show the representative tumors with H&E staining and IHC analysis of erbB2 and erbB3 receptors.

To further explore whether MM-121 holds potential to enhance the efficacy of paclitaxel in breast cancer treatment, we took advantage of a tumor xenograft model established from the trastuzumab-resistant BT474-HR20 cells. For the first set of *in vivo* experiments, when larger tumors (approximately 250 mm^3^) were established, the tumor-bearing mice were treated with either PBS (control), or MM-121 (10 mg/kg) or paclitaxel (15 mg/kg) alone, or with the combinations of MM-121 and paclitaxel. All treatments were carried out by i.p. injection twice a week. We discovered that whereas treatment with MM-121 had no effects on tumor growth, paclitaxel at a dose of 15 mg/kg significantly inhibited tumor growth in this model. Similar inhibitory effects on tumor growth were observed with the combinations of MM-121 and paclitaxel (15 mg/kg) (Additional file 4). For the second set of *in vivo* experiments using smaller tumors (approximately 100 mm^3^), a lower dose of paclitaxel was used to treat the tumor-bearing mice. Although treatment with either MM-121 or paclitaxel (7.5 mg/kg) alone had little effect on tumor growth, their combinations significantly inhibited tumor growth in this xenograft model (Figure 5A). These data suggest that MM-121 enhances low-dose paclitaxel-mediated antitumor activity against erbB2-overexpressing breast cancer in this *in vivo* mouse model. After 3-week (twice/week) treatments, the remaining tumors obtained from the second study were subjected to histology and IHC analyses. Our data revealed that treatment with either MM-121 or paclitaxel (7.5 mg/kg) had no significant effects on tumor cell morphology, tumor mass architecture, and the expression of erbB2/erbB3 receptors (Figure 5B). In contrast, smaller tumor mass and bigger empty spaces among tumor cells were found with the combinatorial treatment. Nonetheless, the remaining tumor cells expressed similar levels of erbB2 and erbB3 (Figure 5B).

### MM-121 in combination with paclitaxel significantly inhibits tumor cell proliferation, reduces expression of Survivin, and promotes more cells undergoing apoptosis in the *in vivo* mouse model

**Figure 6:**
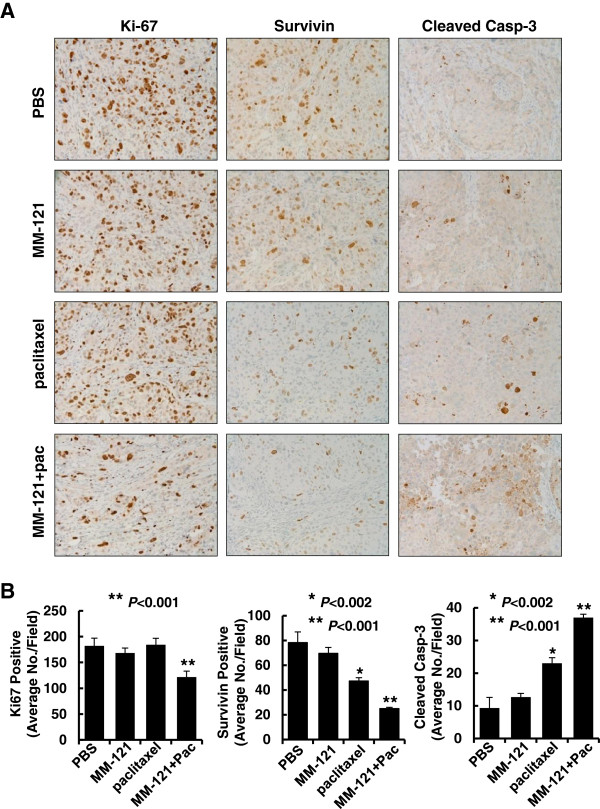
**The combination of MM-121 and paclitaxel significantly inhibits tumor cell proliferation, reduces expression of Survivin, and induces apoptosis in an *in vivo *mouse model.** The tumors obtained from the animal studies described above were evaluated by immunohistochemical (IHC) analysis of Ki-67, Survivin, and cleaved caspase-3. **(A)** Data show the representative images of the immunostainging of Ki-67, Survivin, and cleaved caspase-3. (**B)** The IHC slides were observed by two independent personnel. The tumor cells with positive staining of Ki-67, Survivin, or cleaved caspase-3 were counted from three randomly selected areas in each slide. The three areas were first identified by scanning the entire slide at × 100 magnification, and then the positive-stained cells were counted at × 200 magnification using an Olympus BX40 microscope. The bar graphs show the average of positive staining cells in each field. *Bars* represent SD. ^*^P <0.002 versus control or MM-121. ^**^P <0.001 versus control or single-agent treatment.

Our *in vitro* studies showed that MM-121 significantly enhanced paclitaxel-mediated anti-proliferative/anti-survival effects and facilitated paclitaxel-induced apoptosis in BT474-HR20 cells (Figure 4). We wondered whether the combinations of MM-121 and paclitaxel would exert similar effects on proliferation and apoptosis *in vivo*. The tumor tissues obtained from the animal studies described above were further used for IHC studies on the classic cell proliferative marker, Ki-67, and the apoptosis marker, cleaved caspase-3, as well as the expression of Survivin. The mice treated with MM-121 alone did not have alteration in the number of tumor cells with positive staining for Ki-67, Survivin, and cleaved caspase-3 compared to the control mice (Figure 6A). Although paclitaxel (7.5 mg/kg) alone had no effects on Ki-67 staining, it did significantly decrease the expression of Survivin and induce caspase-3 cleavage in the tumor cells (Figure 6A and B). More importantly, the combinatorial treatments exhibited a significant reduction in the number of tumor cells with positive staining for Ki-67, a striking downregulation of Survivin, and a dramatic increase of the tumor cells with cleaved caspase-3 (Figure 6). These data indicate that MM-121 enhances paclitaxel antitumor activity against erbB2-overexpressing breast cancer via simultaneous inhibition of tumor cell proliferation and induction of apoptosis *in vivo*.

## Discussion

The erbB3 receptor has long been considered as an inactive *pseudokinase*[10,11], although a recent study suggests that erbB3 has weak kinase activity that can trans-autophosphorylate its intracellular region [12]. In order to fully transduce cell signaling, however, erbB3 has to be phosphorylated by its interactive partners; of these, erbB2 is the most important one [33]. It has been well-documented that activation of the erbB3 signaling plays a pivotal role in the development of erbB2-overexpressing breast cancer [13,14]. Although a number of strategies targeting erbB2 are being used in the clinic [34], no erbB3-targeted therapy has been approved for cancer treatment. MM-121 is an erbB3-blocking Ab that is being actively investigated in clinical trials of cancer patients with solid tumors, such as advanced non-small cell lung cancer, colorectal cancer, squamous cell head and neck cancer, and platinum resistant/refractory ovarian cancer [35]. In breast cancer, the therapeutic potential of MM-121 is being tested in patients with estrogen receptor- and/or progesterone receptor-positive and erbB2-negative breast cancers in combination with the aromatase inhibitor exemestane, and in patients with triple-negative or erbB2-negative breast cancers in combination with paclitaxel. To date, no study has been initiated to test the activity of MM-121 in breast cancer patients with erbB2-overexpressing tumors, including those resistant to paclitaxel. Here we provide experimental evidence indicating that MM-121 inactivates erbB3 (Figures 1B and 4A), significantly enhances paclitaxel-induced anti-proliferative/anti-survival effects (Figures 1A, 4B, 5A, and 6), and apoptosis (Figures 2B, 2C, 4C, 4D, and 6) in erbB2-overexpressing breast cancer cells with either medium (SKBR3, SKBR3.neo1, and BT474-HR20) or high erbB3 (SKBR3.B3.1 and SKBR3.B3.2) expression. Our data suggest that MM-121 is efficacious in all erbB2-overexpressing breast cancer cell lines tested. We believe that MM-121 will exhibit therapeutic potential in all erbB2-positive breast cancer patients (not only those with strong erbB3-expressing tumors) as long as the tumors show active erbB3 signaling.

The concentrations of paclitaxel used in the *in vitro* studies (0 to 16 nmol/L or 0 to 32 nmol/L) are much lower than the average steady-state plasma concentrations of paclitaxel (300 to 800 nmol/L) found in patients [36,37]. This difference may be explained by the two very distinct systems. In the 2-dimensional cell culture condition, paclitaxel should easily get into the tumor cells which grow as a monolayer. However, due to the complexity of the human body, some substances in the circulation may attenuate the efficacy of paclitaxel. The presence of fibroblast, microphage, immune cells and others in the tumor mass may block or decrease the drug’s entry into the tumor cells. In addition, because of the heterogeneicity of tumors and their 3-dimensional architecture, drug uptake varies in tumor cells. Thus, the steady-state plasma concentration of paclitaxel may not reflect the drug concentration inside the tumor cells. The data obtained from our animal studies provide further support of this point. It has been reported that administration of 10 mg/kg paclitaxel to mice gives rise to a peak plasma concentration of approximately 3 μmol/L, which gradually and near-linearly declines to 0 at 16 h [38]. This peak concentration of paclitaxel is much higher than that we used in our cell culture studies. However, in the *in vivo* studies, although administration of 15 mg/kg paclitaxel to mice had significant inhibitory effects on tumor growth (Additional file 4), we found no such effect when paclitaxel was used at 7.5 mg/kg (Figure 5A). Thus, both our *in vitro* and *in vivo* data seem to indicate that the ability of MM-121 to enhance the therapeutic efficacy of paclitaxel against erbB2-overexpressing breast cancer may be restricted to the ineffective doses of paclitaxel. Although a recent report shows that as a single agent, neoadjuvant treatment with paclitaxel induces tumor response in 92% of patients with erbB2-overexpressing breast cancer [39], our findings suggest that the presence of MM-121 may convert the tumors from non-responsive to responsive to the lower doses of paclitaxel with less side effects, and therefore merit translational implications in the clinic.

We have reported that the underlying mechanism that elevated expression of erbB3 results in paclitaxel resistance in erbB2-overexpressing breast cancer cells is attributed to PI-3 K/Akt-dependent upregulation of Survivin [27]. Numerous studies indicate that Survivin functions as a critical inhibitor of apoptosis to promote cell survival and proliferation, and regulates mitosis during cell cycle progression [40]. Survivin is selectively expressed in a variety of human malignancies and its overexpression positively correlates with poor prognosis, tumor recurrence and therapeutic resistance [40]. A number of different strategies targeting Survivin, including antisense oligonucleotide and pharmacological inhibitors have been developed and are currently under clinical trials for cancer treatment [40-42]. Our data showed that inactivation of erbB3 signaling with MM-121 specifically downregulated Survivin in our *in vitro* models, and significantly enhanced paclitaxel-induced cytotoxicity and apoptosis in the otherwise resistant breast cancer cells (Figures 1 and 2). In our mouse model, although treatment with MM-121 alone had no significant effects on Survivin expression, MM-121 did dramatically downregulate Survivin when combined with paclitaxel (Figure 6). It is possible that MM-121 at the dose we used only partially inhibits the PI-3 K/Akt signaling *in vivo*, and the inactivation of Akt to such an extent alone could not significantly alter the expression of Survivin. Alternatively, other signaling pathways may also be critical to control Survivin expression in the *in vivo* circumstance. A marked reduction of Survivin was only observed with the combinatorial treatment. In addition, we have found that hMP-RM-1, a humanized version of the anti-erbB3 Ab MP-RM-1 [43], exhibited similar *in vitro* activity to specifically downregulate Survivin in erbB2-overexpressing breast cancer cells (data not shown). Thus, inhibition of erbB3 with blocking Ab may be a novel strategy to target Survivin for cancer treatment. As MP-RM-1 inhibits both ligand-dependent and -independent activation of erbB3 [43], we speculate that hMP-RM-1 might exert more potent antitumor activity than MM-121 against erbB2-overexpressing breast cancer. More detailed studies are needed to confirm our hypothesis.

Elevated expression of Survivin was also observed in the trastuzumab-resistant subline BT474-HR20 and causally led the cells resistant to paclitaxel-induced apoptosis (Additional files 3 and 4). However, we did not find Survivin upregulation in another trastuzumab-resistant subline SKBR3-pool2. These data suggest that elevated expression of Survivin may cause cross-resistance to paclitaxel treatment in some trastuzumab-resistant breast cancers. We believe that profound activation of PI-3 K/Akt signaling, which we observed in BT474-HR20, but not in SKBR3-pool2 cells [28], is the major mechanism contributing to the upregulation of Survivin. At this moment, it remains unclear whether activation of the PI-3 K/Akt signaling by any means, such as *PIK3CA* mutation or phosphatase and tensin homolog (PTEN) deletion in addition to erbB2/erbB3 receptors, would also enhance expression of Survivin in breast cancer cells. The interesting phenomenon of Survivin-mediated cross-resistance to paclitaxel and trastuzumab warrants further investigation.

Activation of the PI-3 K/Akt signaling has been identified as the major determinant of trastuzumab resistance [29]. In addition to inducing paclitaxel resistance, the erbB2/erbB3/PI-3 K/Akt signaling also results in resistance to hormonal therapy [44] and other chemotherapy [45] in breast cancer treatment. Because MM-121 mainly inhibits activation of erbB3 and Akt in erbB2-overexpressing breast cancer cells, it is conceivable to hypothesize that MM-121 may abrogate erbB3 signaling-mediated therapeutic resistance to tamoxifen, trastuzumab, and other chemotherapeutic agents, such as doxorubicin. By taking advantage of the trastuzumab-resistant breast cancer model (BT474-HR20 and SKBR3-pool2), we have discovered that MM-121 is able to overcome trastuzumab resistance and significantly enhance trastuzumab-induced growth inhibition and/or apoptosis *in vitro* and *in vivo* (submitted separately).

## Conclusions

We demonstrate that targeting of erbB3 with the blocking Ab MM-121 significantly enhances paclitaxel antitumor activity against erbB2-overexpressing breast cancer cells in our *in vitro* and *in vivo* models. In those models, MM-121 is active to overcome the resistance to paclitaxel, and such a capability of MM-121 may be restricted to the ineffective doses of paclitaxel. Mechanistically, MM-121 inhibits the PI-3 K/Akt signaling, downregulates Survivin, and subsequently enhances paclitaxel-mediated cytotoxicity and apoptosis *in vitro*. The combinations of MM-121 and paclitaxel significantly inhibit tumor cell proliferation, reduce expression of Survivin, and induce apoptosis *in vivo.* Our data support further studies to explore the therapeutic potential of MM-121 in combination with paclitaxel in breast cancer patients with erbB2-overexpressing tumors.

## Abbreviations

Ab: Antibody; Akt: Protein kinase B; BSA: Bovine serum albumin; DMEM: Dulbecco’s modified Eagle’s medium; EGFR: Epidermal growth factor receptor; ELISA: Enzyme-linked immunosorbent assay; FBS: Fetal bovine serum; H&E: Hematoxylin and eosin; IGF-1R: Insulin-like growth factor-I receptor; IGF-I: Insulin-like growth factor-I; IHC: Immunohistochemistry; i.p.: Intraperitoneal; MAPK: Mitogen-activated protein kinase; MEK: Mitogen-activated protein kinase kinase; MTS: 3-(4,5-dimethylthiazol-2-yl)-5-(3-carboxymethoxyphenyl)-2-(4-sulfophenyl)-2H-tetrazolium, inner salt; P-Atk: Phosphorylated Akt; P-MAPK: Phosphorylated mitogen-activated protein kinase; PARP: Poly(ADP-ribose) polymerase; PI-3K: Phosphoinositide 3-kinase; PTEN: Phosphatase and tensin homolog; RTK: Receptor tyrosine kinase; shRNA: Short-hairpin RNA.

## Competing interests

The authors declare that they have no competing interests. MM-121 was kindly provided by Merrimack Pharmaceuticals, Inc. (Cambridge, MA, USA).

## Authors’ contributions

The authors’ contributions to this research work are reflected in the order shown, with the exception of BL who supervised the research and finalized the report. SW, JH, BC, and HL carried out all the *in vitro* experiments and most of the *in vivo* experiments. SW and BL drafted the manuscript. XY and FL participated in the animal studies. JH, BC, and SE reviewed the IHC slides and counted the positive staining. JT, AT, CKL, and BL conceived of the study, and participated in its design and coordination. All authors read and approved the final manuscript.

## Supplementary Material

Additional file 1: Figure S4 Evaluation of specificity of the antibodies used in the immunohistochemistry (IHC) studies. **(A)** The anti-Ki67 antibody was tested using the tumor xenografts established by the human breast cancer cell line MDA-MB-231 in a nude mouse. The picture was taken at ×100 magnification by an Olympus BX40 microscope. Both positive-staining MDA-MB-231 tumor cells and negative staining of the adjacent mouse lymphocytes are indicated as (+) and (-), respectively. **(B)** The human breast cancer cell line MCF-7, which has no erbB2 expression, and SKBR3, which is a well-known erbB2-overexpressing breast cancer cell line, were used to assess the anti-erbB2 antibody. Both cell lines under normal culture condition were collected, paraffin-embedded, and then followed by the standard procedure of IHC analysis. The pictures were taken at ×200 magnification. **(C)** Tumor sections obtained from the BT474-HR20 tumor xenografts described in the current studies (in the animals without treatment) were used to evaluate the antibodies against erbB3, Survivin, and cleaved caspase-3. All pictures were taken at ×200 magnification. Wheareas the BT474-HR20 tumor cells showed positive staining of erbB3 (+), Survivin (+), and rare cleaved caspase-3 (+), the fibroblast cells (likely from the host) stained negative for erbB3 (-) and the adjacent mouse lymphocytes stained negative for Survivin (-). The majority of the BT474-HR20 tumor cells were negative-stained for cleaved caspase-3 (-), because the animals received no treatment. Click here for file

Additional file 2: Figure S1 SKBR3.B3.1 and SKBR3.B3.2 cell lines are less responsive to paclitaxel-mediated anti-proliferative/anti-survival effects than SKBR3.neo1 cells. SKBR3.neo1, SKBR3.B3.1, or SKBR3.B3.2 cells were plated onto 96-well plates and incubated at 37°C with 5% CO_2_. After 24 h, the culture medium was replaced with 0.1 ml fresh medium containing 0.5% FBS or the same medium containing the indicated concentrations of paclitaxel for another 72 h. The percentages of surviving cells from each cell line relative to controls, defined as 100% survival, were determined by reduction of 3-(4,5-dimethylthiazol-2-yl)-5-(3-carboxymethoxyphenyl)-2-(4-sulfophenyl)-2H-tetrazolium, inner salt (MTS). Bars represent SD. Data are representative of three independent experiments. Click here for file

Additional file 3: Figure S2 The trastuzumab-resistant BT474-HR20 cells have elevated expression of Survivin and are significantly more resistant to paclitaxel-mediated anti-proliferative/anti-survival effects than the parental BT474 cells. **(A)** BT474 and its trastuzumab-resistant subline BT474-HR20 cells in normal culture condition were collected and subjected to western blot analyses of Survivin, Bcl-xL, Mcl-1, or β-actin. **(B)** BT474 or BT474-HR20 cells were plated onto 96-well plates and incubated at 37°C with 5% CO_2_. After 24 h, the culture medium was replaced with 0.1 ml fresh medium containing 0.5% FBS or the same medium containing the indicated concentrations of paclitaxel for another 72 h. The percentages of surviving cells from each cell line relative to controls, defined as 100% survival, were determined by reduction of 3-(4,5-dimethylthiazol-2-yl)-5-(3-carboxymethoxyphenyl)-2-(4-sulfophenyl)-2H-tetrazolium, inner salt (MTS). Bars represent SD. Data are representative of three independent experiments. Click here for file

Additional file 4: Figure S3 Combinations of MM-121 and high-dose paclitaxel exhibit similar activity to high-dose paclitaxel alone to inhibit tumor growth of trastuzumab-resistant breast cancer cells *in vivo*. BT474-HR20 thyc=5?> cells were subcutaneously injected into nude mice to establish tumor xenografts. The tumor-bearing mice (n = 5) received intraperitoneal injections of either PBS, or MM-121 (10 mg/kg), or paclitaxel (15 mg/kg) alone, or both MM-121 and paclitaxel as described in Methods. After four treatments, the mice were euthanized at day 45 post inoculation of tumor cells. The graphs show the tumor growth curves. Bars represent SD. Click here for file
