# Supplementary figures and images for: Therapeutic targeting of erbB3 with MM-121/SAR256212 enhances antitumor activity of paclitaxel against erbB2-overexpressing breast cancer
Source: Breast Cancer Res. 2013 Oct 29;15(5):R101. doi: 10.1186/bcr3563 (PMC3978722; doi:10.1186/bcr3563)

## Slide 1
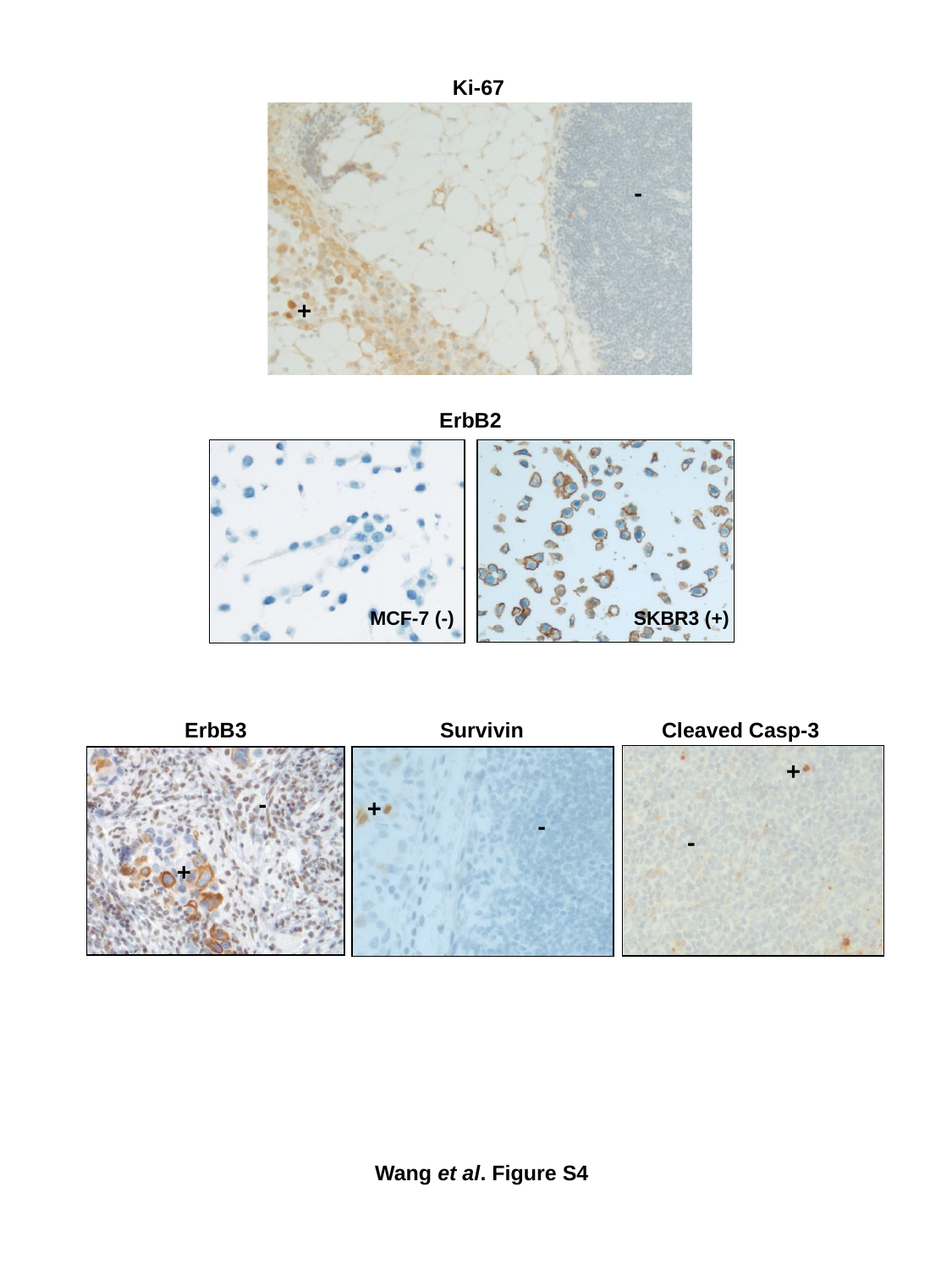

Ki-67
-
+
ErbB2
MCF-7 (-)
SKBR3 (+)
ErbB3
Survivin
Cleaved Casp-3
+
-
+
-
-
+
Wang et al. Figure S4

Supplement: Additional file 1: Figure S4 — Evaluation of specificity of the antibodies used in the immunohistochemistry (IHC) studies. (A) The anti-Ki67 antibody was tested using the tumor xenografts established by the human breast cancer cell line MDA-MB-231 in a nude mouse. The picture was taken at ×100 magnification by an Olympus BX40 microscope. Both positive-staining MDA-MB-231 tumor cells and negative staining of the adjacent mouse lymphocytes are indicated as (+) and (-), respectively. (B) The human breast cancer cell line MCF-7, which has no erbB2 expression, and SKBR3, which is a well-known erbB2-overexpressing breast cancer cell line, were used to assess the anti-erbB2 antibody. Both cell lines under normal culture condition were collected, paraffin-embedded, and then followed by the standard procedure of IHC analysis. The pictures were taken at ×200 magnification. (C) Tumor sections obtained from the BT474-HR20 tumor xenografts described in the current studies (in the animals without treatment) were used to evaluate the antibodies against erbB3, Survivin, and cleaved caspase-3. All pictures were taken at ×200 magnification. Wheareas the BT474-HR20 tumor cells showed positive staining of erbB3 (+), Survivin (+), and rare cleaved caspase-3 (+), the fibroblast cells (likely from the host) stained negative for erbB3 (-) and the adjacent mouse lymphocytes stained negative for Survivin (-). The majority of the BT474-HR20 tumor cells were negative-stained for cleaved caspase-3 (-), because the animals received no treatment. [file bcr3563-S1.ppt]

## Slide 1
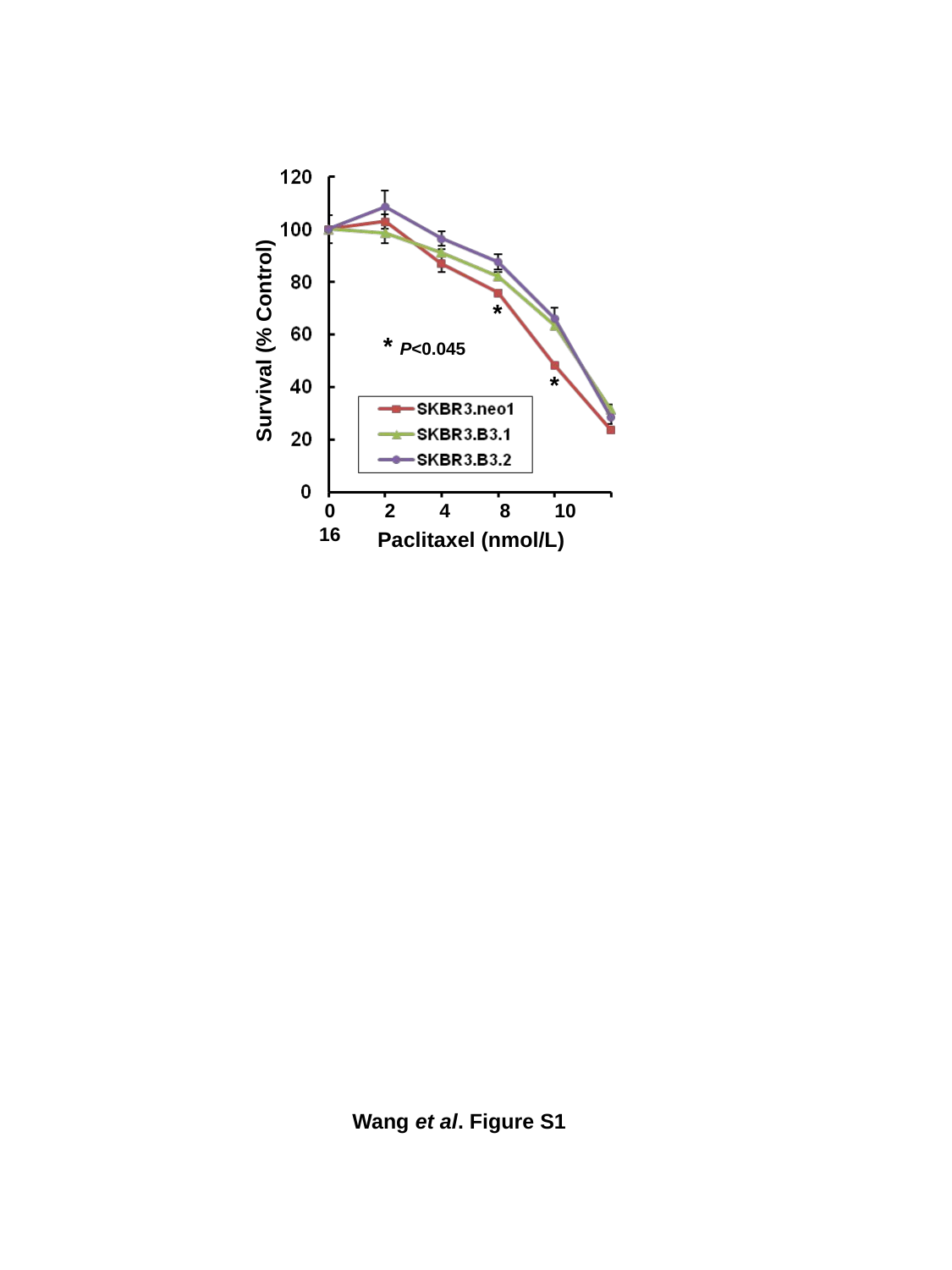

*
Survival (% Control)
* P<0.045
*
 0 2 4 8 10 16
Paclitaxel (nmol/L)
Wang et al. Figure S1

Supplement: Additional file 2: Figure S1 — SKBR3.B3.1 and SKBR3.B3.2 cell lines are less responsive to paclitaxel-mediated anti-proliferative/anti-survival effects than SKBR3.neo1 cells. SKBR3.neo1, SKBR3.B3.1, or SKBR3.B3.2 cells were plated onto 96-well plates and incubated at 37°C with 5% CO2. After 24 h, the culture medium was replaced with 0.1 ml fresh medium containing 0.5% FBS or the same medium containing the indicated concentrations of paclitaxel for another 72 h. The percentages of surviving cells from each cell line relative to controls, defined as 100% survival, were determined by reduction of 3-(4,5-dimethylthiazol-2-yl)-5-(3-carboxymethoxyphenyl)-2-(4-sulfophenyl)-2H-tetrazolium, inner salt (MTS). Bars represent SD. Data are representative of three independent experiments. [file bcr3563-S2.ppt]
