# Supplementary material for: Therapeutic targeting of erbB3 with MM-121/SAR256212 enhances antitumor activity of paclitaxel against erbB2-overexpressing breast cancer
Source: Breast Cancer Res. 2013 Oct 29;15(5):R101. doi: 10.1186/bcr3563 (PMC3978722; doi:10.1186/bcr3563)
Supplement: Additional file 3: Figure S2 — The trastuzumab-resistant BT474-HR20 cells have elevated expression of Survivin and are significantly more resistant to paclitaxel-mediated anti-proliferative/anti-survival effects than the parental BT474 cells. (A) BT474 and its trastuzumab-resistant subline BT474-HR20 cells in normal culture condition were collected and subjected to western blot analyses of Survivin, Bcl-xL, Mcl-1, or β-actin. (B) BT474 or BT474-HR20 cells were plated onto 96-well plates and incubated at 37°C with 5% CO2. After 24 h, the culture medium was replaced with 0.1 ml fresh medium containing 0.5% FBS or the same medium containing the indicated concentrations of paclitaxel for another 72 h. The percentages of surviving cells from each cell line relative to controls, defined as 100% survival, were determined by reduction of 3-(4,5-dimethylthiazol-2-yl)-5-(3-carboxymethoxyphenyl)-2-(4-sulfophenyl)-2H-tetrazolium, inner salt (MTS). Bars represent SD. Data are representative of three independent experiments. [file bcr3563-S3.ppt]

## Slide 1
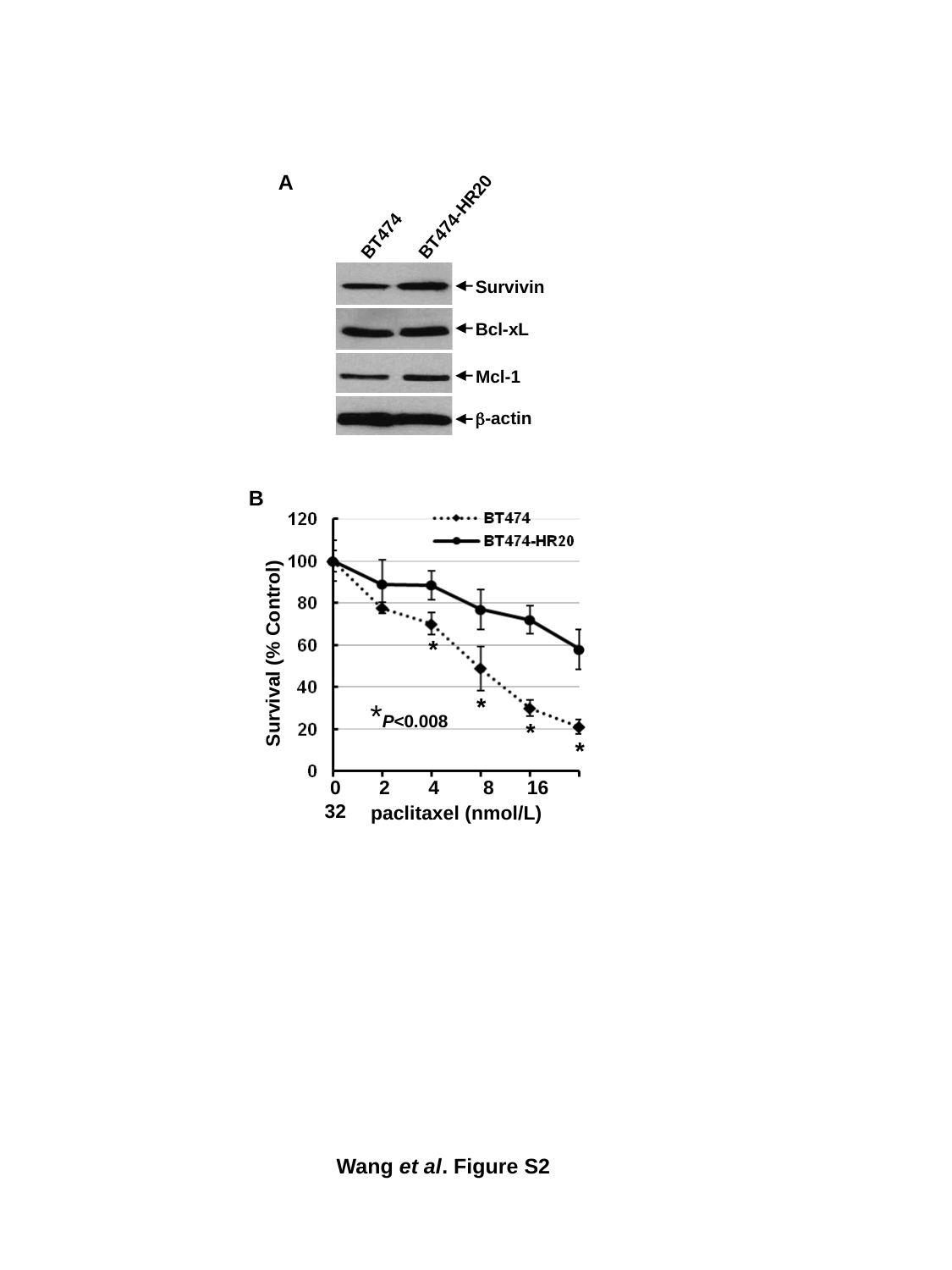

BT474-HR20
BT474
Survivin
Bcl-xL
Mcl-1
β-actin
A
B
*
Survival (% Control)
*
*P<0.008
*
*
 0 2 4 8 16 32
paclitaxel (nmol/L)
Wang et al. Figure S2
