# Supplementary material for: Therapeutic targeting of erbB3 with MM-121/SAR256212 enhances antitumor activity of paclitaxel against erbB2-overexpressing breast cancer
Source: Breast Cancer Res. 2013 Oct 29;15(5):R101. doi: 10.1186/bcr3563 (PMC3978722; doi:10.1186/bcr3563)
Supplement: Additional file 4: Figure S3 — Combinations of MM-121 and high-dose paclitaxel exhibit similar activity to high-dose paclitaxel alone to inhibit tumor growth of trastuzumab-resistant breast cancer cells in vivo. BT474-HR20 thyc=5?> cells were subcutaneously injected into nude mice to establish tumor xenografts. The tumor-bearing mice (n = 5) received intraperitoneal injections of either PBS, or MM-121 (10 mg/kg), or paclitaxel (15 mg/kg) alone, or both MM-121 and paclitaxel as described in Methods. After four treatments, the mice were euthanized at day 45 post inoculation of tumor cells. The graphs show the tumor growth curves. Bars represent SD. [file bcr3563-S4.ppt]

## Slide 1
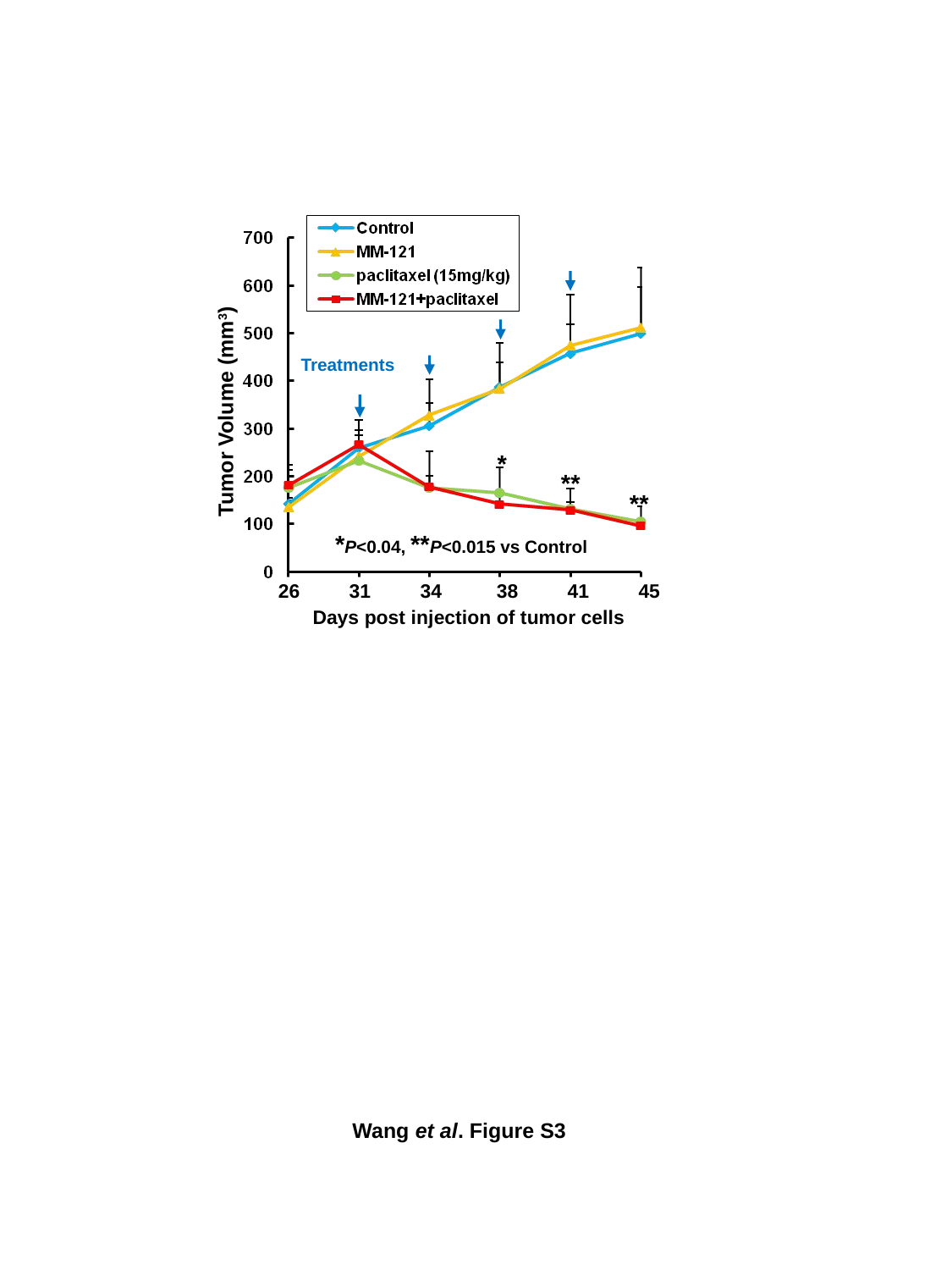

Treatments
Tumor Volume (mm3)
*
**
**
*P<0.04, **P<0.015 vs Control
26 31 34 38 41 45
Days post injection of tumor cells
Wang et al. Figure S3
